# Notes on Preparations for the Sick

**Published:** 1891-12

**Authors:** 


### Notes on Preparations for the Sick.

**Quinine Tabloids. Cascara Sagrada Tabloids. Charcoal Tabloids.**—BURROUGHS, WELLCOME & CO., London.

These tabloids are excellent preparations, and are a most convenient and efficient form for the administration of these drugs.

The quinine are prepared with the bisulphate, which is twenty-four times more soluble than the ordinary sulphate. The crystals are reduced to a fine powder, and compressed in such a way as to disintegrate in the stomach within a minute from the time they are swallowed. A tabloid in a glass of water will be completely disintegrated in less than two minutes. The tabloids possess all the advantages of a solution as regards rapidity of action, whilst they are most portable, and are easily swallowed.

While it is well known that there is much difference in the effect produced by cascara sagrada upon different persons, this difference would appear attributable in a measure to differences in the products used. Examination of various preparations of cascara sagrada in the market reveals that they vary greatly in strength. Recently a report, published in America, showed that in 58 cases of constipation, the drug was efficient in 44, and that it was found desirable to diminish rather than increase the dose with the lapse of time. These tabloids are coated with pure sugar, and hence are not disagreeable to take. The usual dose of cascara sagrada tabloids is one or two at bedtime, or one after each meal. They are admirably suited for continuous gentle medication, and are soluble. Each tabloid contains 2 grains of dry extract of cascara, prepared from selected and seasoned bark.

The makers state that for a long time they have prepared tabloids which are intended to readily disintegrate in the stomach when swallowed with water. Pure willow charcoal tabloids are the latest addition to the already extensive list. Each contains 5 grains of pure willow charcoal with a small quantity of sugar. The tabloid is not unpalatable if allowed to slowly dissolve on the tongue like a lozenge. It may, however, be swallowed intact with a little water. When it reaches the stomach it will readily disintegrate to a fine powder. Many physicians have been debarred from prescribing this drug on account of its obnoxious appearance when given suspended in mucilage as a mixture, or in the form of biscuits. Charcoal has not been a popular drug on account of the difficulty in taking a sufficient dose, but in the tabloid form there will be no such difficulty of administration, and the utility of the drug as an intestinal antiseptic is undoubtedly great.

---

**Liquor Papain Co.**—A. W. Griffin, Bath.

The composition of this fluid is as follows:—

| | |
|---|---|
| Papain Finkler . . . | grs. ij. |
| Pepsina fortissima . . . | grs. ij. |
| Acidulated glycerine . . . | ʒj. |

It should be a very powerful digestive, and likely to be of service to many of our patients who require artificial aids. The pepsine used is forty times stronger than the B.P. form. It is intended that one or two teaspoonfuls should be taken in water after a meat meal.

---

**Digestive Meal.**—Gregory & Son, Bristol.

This meal consists of wheat (the whole grain) macerated with a solution of malt, and dried at a medium temperature. It contains: —

| | |
|---|---|
| Flesh-forming elements . | 24.5 per cent. |
| Respiratory ,, . | 72.5 ,, |
| Bone-forming ,, . | 3.0 ,, |

It forms an easily-digested and highly-nutritious food, is easily cooked, it makes excellent light puddings, and is worthy of trial for infants and invalids.

---

**Ozonine.**—Dorset Soda Water Company, Weymouth.

This effervescing, non-alcoholic beverage is described as "the greatest health restorer ever placed before the public." It is a very pleasant drink, with a flavour of orange, effervescing well, mounted in champagne bottles, and intended to be taken from champagne glasses; but its cost is less than one-twelfth that of a good brand of champagne. We presume that the name is intended to suggest the idea that this beverage contains bottled-up sea breezes, and will enable those who drink it to dispense with the usual summer holiday at the seaside, an advantage to the pocket as well as to the health.

---

**Cascara Cordial. Pepsin Cordial. Normal Liquid Ergot. Glycerin Suppositories.**—Parke, Davis and Co., Detroit.

Cascara sagrada, the bark of the rhamnus purshiana of Northern California and Oregon, first introduced by Messrs. Parke, Davis & Co., and so named by them, has now become so familiar as to need no recommendation. It may be looked upon as "the perfect laxative," and it rarely fails to give a satisfactory result in cases where a steady intestinal stimulant is habitually required. In one or other of its numerous and varied preparations, it has almost superseded many of the older laxatives: cordials, chocolates, and tabloids having taken the place of pills and castor oil. The elixir of the bark, called by its originators "Cascara Cordial," is in every way a satisfactory and trustworthy preparation.

Pepsin Cordial is an elegant preparation of pepsine in solution in combination with aromatics. It is one-third the strength of saccharated pepsine, and will dissolve fifteen times its weight of coagulated egg-albumin. Of the Liquid Pepsines, this appears to be one of the most active, most permanent, and most palatable. They are a great improvement on the older hygroscopic and nauseous powders.

Normal Liquid Ergot is an assayed preparation of standard and uniform strength, one cubic centimetre being equivalent to one gramme of the drug. This fluid appears to be one of the most satisfactory preparations of ergot yet prepared, and it has given excellent results when used for hypodermic injection.

The Glycerin Suppositories have 95 per cent. of glycerine, each containing three grammes. They are of convenient shape, easily introduced, and are a very efficient substitute for the more troublesome glycerine enema.

Messrs. Burgoyne & Co. are the London Agents for these preparations.

